# Circulating Fibroblast Growth Factor 21 is Associated with Diastolic Dysfunction in Heart Failure Patients with Preserved Ejection Fraction

**DOI:** 10.1038/srep33953

**Published:** 2016-09-21

**Authors:** Ruey-Hsing Chou, Po-Hsun Huang, Chien-Yi Hsu, Chun-Chin Chang, Hsin-Bang Leu, Chin-Chou Huang, Jaw-Wen Chen, Shing-Jong Lin

**Affiliations:** 1 Division of Cardiology, Department of Medicine Taipei Veterans General Hospital, Taipei, 11217, Taiwan; 2 Cardiovascular Research Center, Taipei Veterans General Hospital, Taipei, 11217, Taiwan; 3 Institute of Clinical Medicine, National Yang-Ming University, Taipei, 11217, Taiwan; 4 Department of Internal Medicine, College of Medicine, Taipei Medical University, Taipei, 11031, Taiwan; 5 Division of Cardiology and Cardiovascular Research Center, Department of Internal Medicine, Taipei Medical University Hospital, Taipei, 11031, Taiwan; 6 Healthcare and Management Center Taipei Veterans General Hospital, Taipei, 11217, Taiwan; 7 Department of Medical Education Taipei Veterans General Hospital, Taipei, 11217, Taiwan; 8 Institute of Pharmacology, National Yang-Ming University, Taipei, 11217, Taiwan; 9 Department of Medical Research Taipei Veterans General Hospital, Taipei, 11217, Taiwan; 10 Division of Clinical Research Taipei Veterans General Hospital, Taipei, 11217, Taiwan

## Abstract

Fibroblast growth factor 21 (FGF21), a polypeptide ligand promoted glucose homeostasis and lipids metabolism, was recently reported to attenuate cardiac hypertrophy. The aim of this study was to investigate the impact of FGF21 in diastolic heart failure. Subjects admitted for coronary angiogram were screened for heart failure, and those with left ventricular (LV) ejection fraction < 45% were excluded. Diastolic dysfunction was defined as functional abnormalities that exist during LV relaxation and filling by echocardiographic criteria. Plasma levels of FGF21 and N-terminal Pro-Brain Natriuretic Peptide (NT-pro-BNP) were determined. All patients were followed up for 1 year, or till the occurrence of heart failure readmission or death. Totally 95 patients with diastolic dysfunction and 143 controls were enrolled. Circulating FGF21 level was correlated with echocardiographic parameters of diastolic function and LV end-diastolic pressure (LVEDP). In multivariate logistic analysis, FGF21 was significantly associated with diastolic dysfunction, either identified by echocardiographic criteria (odds ratio: 2.97, *p* = 0.012) or confirmed with LVEDP level (odds ratio: 3.73, *p* = 0.030). Both plasma FGF21 (log rank *p* < 0.0001) and NT-pro-BNP levels (log rank *p* = 0.0057) showed good *p*redictive power to the 1-year adverse cardiac events. This finding suggested FGF21 could be involved in the pathophysiology of diastolic heart failure.

Heart failure is a severe clinical syndrome involving dysregulation between the autonomic, neuroendocrine and immune systems, which continues to be a major health problem worldwide. Heart failure has classically been considered to be a clinical syndrome associated with impaired left ventricular (LV) systolic function. There is growing recognition that among patients with clinical symptoms associated with heart failure, approximately half the patients have preserved LV systolic function, known as heart failure with preserved LV ejection fraction (HFpEF) or diastolic heart failure^1^. Recent epidemiological reports indicated that the prevalence of HFpEF patients is increasing^2^, and the overall prognosis is comparable to heart failure with reduced left ventricular ejection fraction (HFrEF)^1^. Although therapeutic strategies and the survival rate of HFrEF patients have greatly improved over the past years, mortality remains high in diastolic heart failure patients^1^.

Fibroblast growth factors (FGFs) are secreted proteins with diverse metabolic functions, and act as autocrine, paracrine^3^, and endocrine hormones^4^. Increasing evidence suggests that some FGFs act as cardiomyokines, and play an important role in cardiovascular remodeling^5^. FGF21, the 21^st^ member of the FGF family, is discovered as a new type of cytokine that regulates glucose and lipid metabolism^4^. Predominantly produced by liver, FGF21 was initially known as a hepatic endocrine factor that promotes thermogenic activity and modulates lipid metabolism^6^. FGF21 binds to the FGF receptor (FGFR) and β-Klotho^7^, and acts as an adipokine to promote glucose uptake in adipocytes^8^ and regulates insulin sensitivity^9^. Although cardiac FGF21 secretion is lower than hepatic FGF21 secretion, FGF21 is stimulated in response to diverse cardiac stresses^10^. Experimental studies demonstrated that FGF21 prevented cardiac hypertrophy by activating silent information regulator 1 (Sirt1) signaling through the activation of FGFR1c with β-Klotho as a co-receptor^10^. Moreover, deletion of FGF21 was found to contribute to more severe myocardial damage^10^,^11^, and administration of FGF21 protected the mice from ventricular hypertrophy^10^ and adverse cardiac remodeling after myocardial infarction^12^. However, most of the reports supporting the association between FGF21 and cardiomyopathy were obtained from animal data. Clinical studies on FGF21 and heart failure in human subjects are limited. As such, we designed this study to investigate the role of circulating FGF21 in diastolic heart failure, and compare the predictive performance of FGF21 with N-terminal Pro-Brain Natriuretic Peptide (NT-pro-BNP).

## Results

### Baseline Characteristics

A total of 95 diastolic heart failure patients and 143 controls were enrolled for analysis. Table 1 summarizes the demographic and clinical characteristics of study subjects. Patients with diastolic heart failure were older, had more occurrences of hypertension, diabetes and multiple vessel disease, more severe heart failure symptoms, and were taking more angiotensin-converting-enzyme inhibitors, beta blockers, and diuretics. Moreover, diastolic heart failure patients have decreased hemoglobin, reduced renal function, and higher fasting blood sugar concentration.

### FGF21 and NT-pro-BNP in Diastolic Heart Failure

As shown in Table 2, plasma levels of FGF21 and NT-pro-BNP were significantly increased in subjects with diastolic dysfunction. Echocardiography demonstrated that diastolic heart failure patients had greater left atrial (LA) dimension, LV mass index (LVMI), E/e′ ratio, and higher right ventricular systolic pressure (RVSP) level. Cardiac catheterization data revealed that patients with diastolic heart failure had higher LV end-diastolic pressure (LVEDP) level and greater pulse pressure, and there was no difference in LV ejection fraction (LVEF) between the two groups.

**Figure 1:**
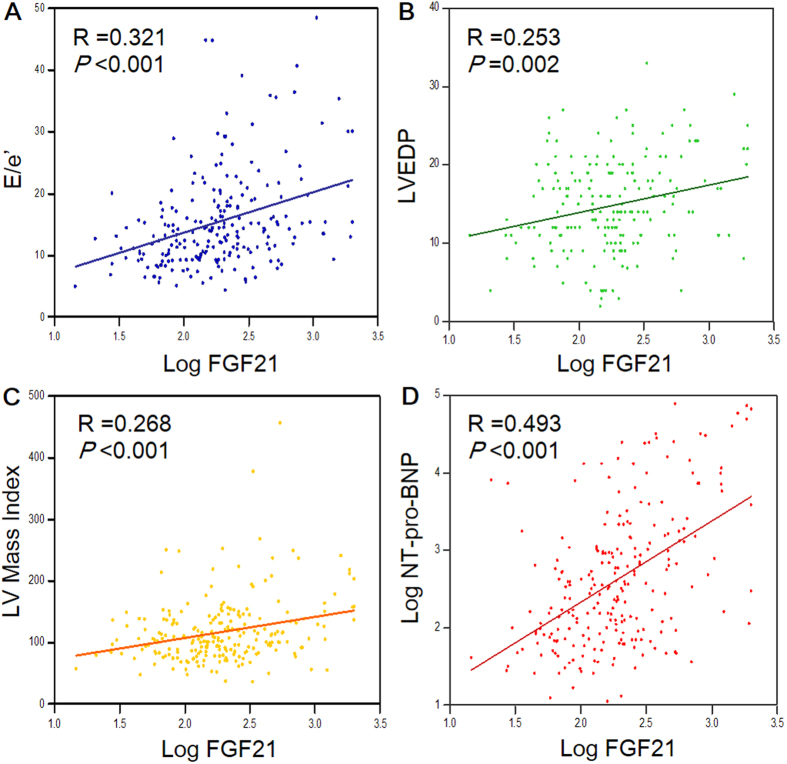
*Scatter plots* and correlation coefficients between log FGF21 and (1A) E/e’ ratio (1B) LVEDP level (1C) LV mass index **(1D)** log NT-pro-BNP values. Spearman’s correlation analysis was performed. (LV = left ventricular, LVEDP = left ventricular end-diastolic pressure).

Both FGF21 and NT-pro-BNP levels presented significant positive correlation with hypertension, diabetes, severity of heart failure, coronary artery disease (CAD), peripheral artery disease, LA dimension, LVMI, E/e′ ratio, and LVEDP; negatively associated with hemoglobin level, estimated glomerular filtration rate (eGFR), and central diastolic blood pressure (DBP) (Table 3). Moreover, circulating FGF21 level was significantly associated with NT-pro-BNP concentration (r = 0.493, *p* < 0.001). Scatter plots of the correlation between FGF21, NT-pro-BNP, E/e′ ratio, LVEDP, and LVMI are presented in Fig. 1.

We further performed linear regression analysis to clarify the association between various parameters, FGF21, NT-pro-BNP and E/e′ ratio, and LVEDP (Supplement Table 1). In univariate analysis, log FGF21, log NT-pro-BNP, age, gender, multiple vessel disease (MVD), eGFR, and fasting glucose levels were associated with E/e′ ratio. Additionally, all the variables, except for gender and MVD were significantly associated with LVEDP measured by cardiac catheterization. In the first multivariate model (model 1), after having adjusted age and gender, both log FGF21 and log NT-pro-BNP were still significantly associated with E/e′ and LVEDP. But in the second multivariate model (model 2), which adjusted all statistically significant variables in univariate analysis, the association between log NT-pro-BNP and LVEDP had become insignificant. In multivariate linear regression analysis, log FGF21 (*p* = 0.011) showed better association with LVEDP than log NT-pro-BNP (*p* = 0.082).

Logistic regression analysis was also performed to investigate the association between FGF21 and diastolic dysfunction (Table 4). We analyzed subjects by two different definitions of diastolic dysfunction in this step. The broad definition included all subjects having met the echocardiographic criteria of diastolic dysfunction, whereas the strict definition included only subjects having met the echocardiographic criteria and with LVEDP > 16 mmHg at the same time^13^. In univariate analysis, log FGF21, log NT-pro-BNP, age, gender, MVD, and eGFR were significantly associated with diastolic dysfunction diagnosed by echocardiography. Even after adjusting age and gender (model 1), both log FGF21 and log NT-pro-BNP retained significant association with diastolic dysfunction. However, under the strict definition of diastolic dysfunction, the result of log NT-pro-BNP became statistically insignificant after adjusting all statistically significant variables in univariate analysis (model 2). Using multivariate logistic regression analysis, log FGF21 was significantly associated with diastolic dysfunction, either diagnosed by echocardiographic criteria alone (odds ratio: 2.97, *p* = 0.012) or confirmed with the LVEDP level (odds ratio: 3.73, *p* = 0.030). Similar results were also noted when grouping subjects according to their FGF21 level in the logistic regression analysis (Supplement Table 2).

### Predictive Power and Prognostic Value of FGF21

**Figure 2:**
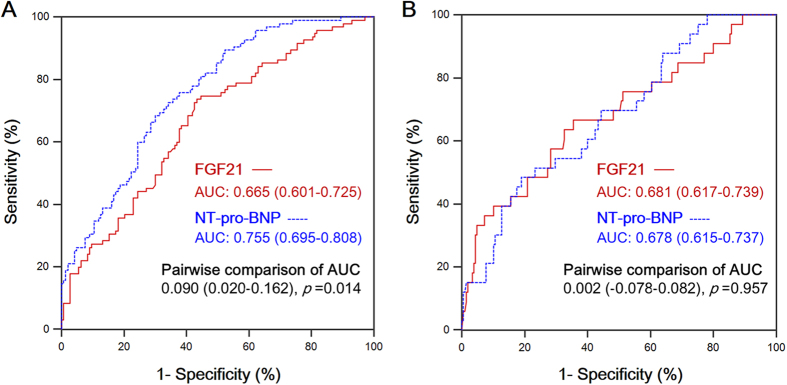
Pairwise comparison of the ROC curve between FGF21 and NT-pro-BNP in predicting diastolic dysfunction. (**2A**) Diastolic dysfunction was diagnosed by echocardiogram alone. **(2B)** Diastolic dysfunction was diagnosed by echocardiogram, and further confirmed with the LVEDP level (LVEDP > 16 mmHg). The statistical significance of the difference between 2 ROC curves was evaluated with the method of *DeLong et al.*^38^. (LVEDP = left ventricular end-diastolic pressure, AUC = area under ROC curve).

The receiver operating characteristic (ROC) curves of FGF21 and NT-pro-BNP in predicting diastolic dysfunction were presented in Fig. 2, and pairwise comparison between areas under ROC curves (AUCs) were performed. When diastolic dysfunction was diagnosed by echocardiography alone (Fig. 2A), NT-pro-BNP showed a somewhat better discriminatory performance than FGF21 (*p* = 0.014). When diastolic dysfunction was diagnosed by echocardiography as well as the cardiac-catheterization results (Fig. 2B), the difference between FGF21 and NT-pro-BNP did not achieve statistical significance (*p* = 0.957). Taking LVEDP into consideration, the predictive power of FGF21 was non-inferior to NT-pro-BNP in assessment of diastolic dysfunction.

**Figure 3:**
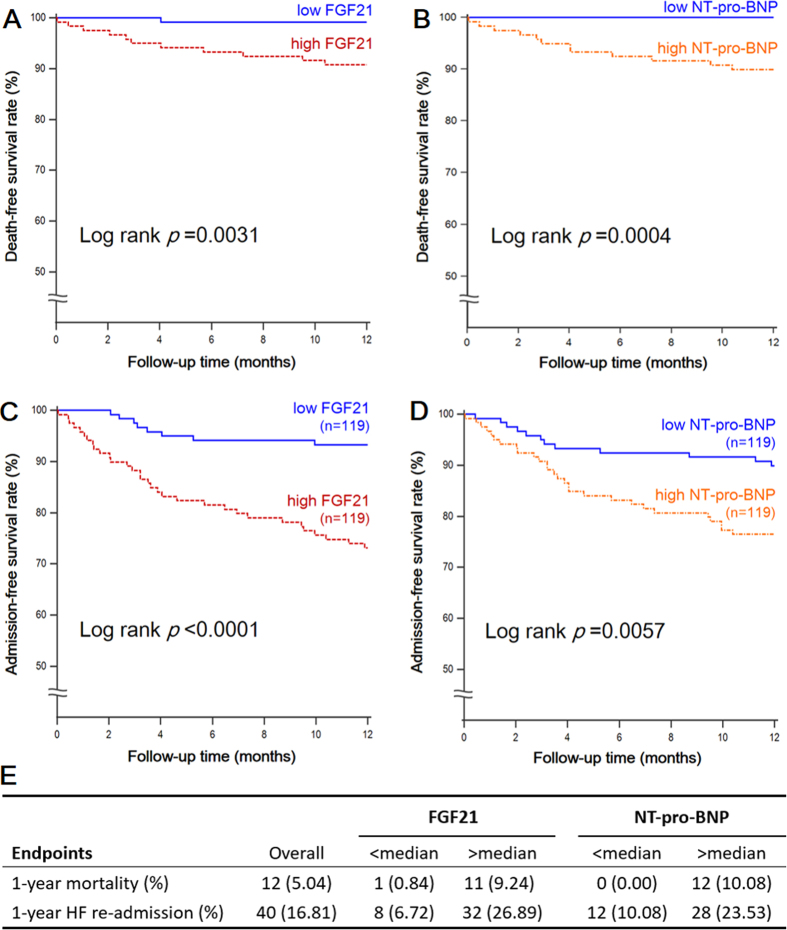
Kaplan-Meier curves of freedom from 1-year mortality (3A-3B) and 1-year readmission events (3C-3D) in patients grouped by FGF21 and NT-pro-BNP levels. Patients with serum FGF21/ NT-pro-BNP level higher than the median were defined as high FGF21/ NT-pro-BNP group. Incidence of death and readmission events is presented in 3E.

A total of 40 cases of heart failure readmission (16.81%) were identified after following up for 1 year, including 12 cases of death (5.04%). The average follow-up time was 10.72 ± 3.17 months. Patients were divided into two equal groups according to their circulating FGF21 or NT-pro-BNP concentrations. The Kaplan–Meier analysis demonstrated a significant difference in 1-year mortality (log rank *p* = 0.0031) and 1-year heart failure readmission events (log rank *p* < 0.0001) between subjects grouped by FGF21 value (Fig. 3). Patients in the high-FGF21 group had a significantly lower adverse event-free survival rate. The Kaplan–Meier analysis grouped by NT-pro-BNP value also showed similar results. Both FGF21 and NT-pro-BNP concentrations had good prognostic value to the 1-year adverse cardiac events in patients with preserved ejection fraction heart failure.

## Discussion

To the best of our knowledge, this is the first study to show that increased plasma FGF21 level was associated with diastolic dysfunction in human subjects. Moreover, circulating FGF21 was non-inferior to NT-pro-BNP in predicting the presence of diastolic dysfunction, as well as predicting 1-year adverse cardiac events in patients with diastolic heart failure. These findings provide indirect evidence of FGF21’s involvement in the cardiac remodeling and bring new insights into the pathophysiological roles of FGF21, which might be a premising therapeutic target of both metabolic and cardiovascular diseases.

HFpEF, also known as diastolic HF, is diagnosed by clinical HF symptoms, and evidence of diastolic dysfunction was mainly accessed by cardiac echocardiography or cardiac catheterization^14^,^15^. HFpEF is quite common that about half of patients with HF symptoms have normal to near-normal LVEF^16^, but its impact on long-term survival is non-inferior to HFrEF^1^,^2^. A verity of comorbidities were reported to be associated with HFpEF, including hypertension, diabetes, chronic kidney disease, CAD^17^, and sleep apnea^18^. Although the survival rate of HFrEF patients have much improved over the past years, mortality remains high in diastolic heart failure and continues to lack a defined treatment strategy^15^. It is thus important to identify new therapeutic targets in patients with diastolic heart failure.

FGF21 is a novel polypeptide ligand that has been shown to play a pivotal role in the regulation of glucose homeostasis and lipid metabolism. After binding to the FGF receptor and β-Klotho, FGF21 activates the Mitogen-Activated Protein Kinase (MAPK) signaling pathway^19^, and enhances glucose uptake and ketogenesis. The heart was traditionally not regarded as a target or source of FGF21 because of modest expression of FGF21 and β-Klotho^20^. However, emerging evidence suggests that the cardiomyocyte secreted FGF21 as an autocrine factor to protect the heart from adverse cardiac remodeling^3^,^5^. Cultured cardiomyocytes were found to secrete FGF21 in response to isoproterenol infusion^10^. Based on these findings, the potential association between FGF21 and diastolic function should be tested in patients with HF. Our study first demonstrated that increased FGF21 was noted in HF patients with diastolic dysfunction, which suggests that FGF21 could be stimulated in the process of diastolic dysfunction.

Several possible rationales could explain the association between FGF21 and diastolic dysfunction. In condition of HFpEF, myocardiocytes may secret FGF21 to protect the heart from adverse cardiac remodeling and leads to a modest rise of serum FGF21 level. FGF21 could directly act on cardiomyocytes to suppress the inflammatory pathway and attenuate cardiac hypertrophy^10^. FGF21-deficient mice were found to exhibit significant cardiac hypertrophy after isoproterenol infusion, which could be prevented by exogenous administration of FGF21^10^. In the phenylephrine-induced-LVH model, Planavila *et al*. found a significant elevation of pro-inflammatory markers and reduction of fatty acid oxidation in the cardiomyocytes of FGF21-KO mice, which could be reversed by providing FGF21 supply^10^. FGF21 may act as an endocrine as well as an autocrine factor to suppress oxidative stress and prevent cardiac hypertrophy. Additionally, FGF21 could prevent the heart from pathological remodeling after myocardial infarction (MI)^12^, which was identified as a common cause of diastolic dysfunction. In the MI model caused by left anterior descending coronary ligation, Joki *et al*. found that mice treated with FGF21 had fewer cardiomyocyte apoptosis, higher capillary density in the infarcted border zone, and better LV systolic function after MI event^12^. Furthermore, FGF21 was reported to be involved in the regulation of cardiac lipid metabolism^21^, which suggests to play a critical role in diabetic cardiomyopathy^22^. Yan *et al*. showed that the deletion of FGF21 would promote lipid uptake and accumulation in cardiomyocytes, ultimately leading to adverse cardiac remodeling in the diabetic mice^11^. Increased serum FGF21 concentration may also be a compensatory response to comorbid metabolic diseases that caused HFpHF, such as diabetes and obesity, which caused massive secretion of FGF21 from liver and adipose tissue. Nevertheless, despite all these *in vivo* and *in vitro* studies, clinical evidence supporting FGF21’s effect on cardiac remodeling remains limited, especially in the population of HFpEF.

Recent clinical studies had reported the correlation between serum FGF21 and cardiovascular disease, such as hypertension^23^, coronary artery disease^24^, and atrial fibrillation;^25^ but very few reports had investigated the relationship between FGF21 and heart failure. Planavila *et al*. conducted a cross-sectional study, which enrolled 6 patients of dilated cardiomyopathy waiting for transplantation and 10 health donors^26^. Patient with end-stage HF had presented a significantly higher serum FGF21 concentration, as well as FGF21 mRNA expression in cardiac tissues. Though lacking the biochemistry profiles generated from cardiac tissues, our study provided detailed hemodynamic data and clinical outcomes to investigate the association between circulating FGF21 and diastolic dysfunction in 238 patients of HFpEF. In our study, we showed the association between circulating FGF21, LV hypertrophy, and diastolic dysfunction in heart failure subjects. Although our results did not support FGF21 to replace the role of NT-pro-BNP, the strong association between FGF21 and diastolic dysfunction supported the findings of previous animal studies and provided us novel insight of the how metabolic regulators affect the progression of early-stage heart failure.

There were several limitations in our study. First of all, it was a retrospective study with relatively small case numbers, which limited the generalization of its findings. Enrolled patients with HFpEF were significantly older, which might imply higher levels of serum FGF21 according to the previous study^27^. Second, there might be selection bias in patient enrollment. Because we selected HF cases from patients admitted for elective coronary angiogram, the symptoms of HF were relatively mild and stable in this population. In addition, we had excluded subjects with atrial fibrillation, whom were at high-risk of diastolic dysfunction and usually had poorer outcome^28^. Compared to the population-based studies that enrolled patients hospitalized for HFpEF, our patients presented a much lower 1-year mortality rate. And it’s a pity that FGF21 levels were only measured in a single point in patients with HFpEF. Data about the change of FGF21 concentration at the time of heart failure progression were absent. Finally, the echocardiography and cardiac catheterization studies were not performed at the same time, which might lead to inconsistency of the results. Nevertheless, because all enrolled patients were with stable HF conditions, their LV filling pressures were supposed to be constant during hospitalization.

In conclusion, this study showed that circulating FGF21 is associated with diastolic dysfunction and 1-year adverse events in patients with diastolic heart failure. These findings suggest that FGF21 may be involved in the pathophysiology of myocardial remodeling and could be a potential therapeutic target in the treatment of heart failure.

## Methods

### Study Population

**Figure 4:**
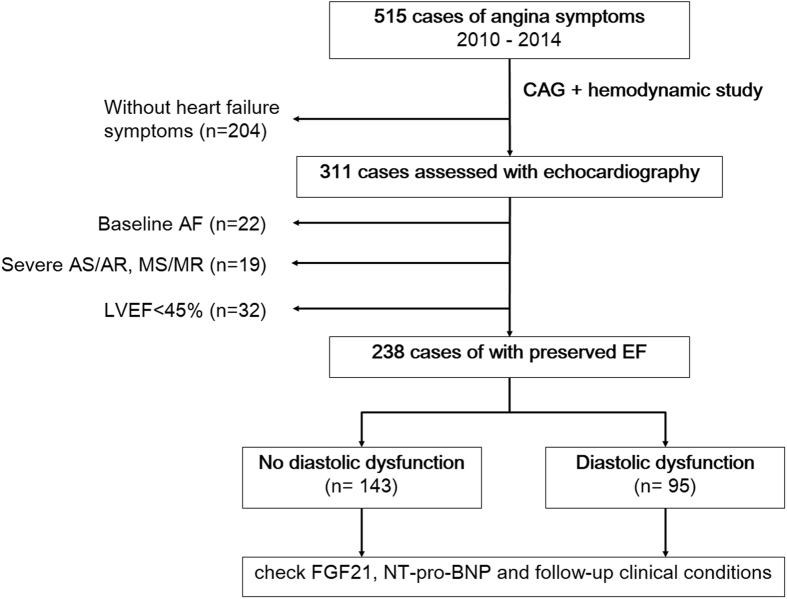
Flowchart of patient enrollment. (CAG = coronary angiography, AF = atrial fibrillation, AS = aortic stenosis, AR = aortic regurgitation, MS = mitral stenosis, MR = mitral regurgitation, LVEF = left ventricular ejection fraction).

From February 2010 to December 2014, 515 subjects who had stable CAD admitted for elective coronary angiogram at Taipei Veterans General Hospital were screened. Pre-procedure laboratory exam and cardiac catheterization were performed for each patient. Patients older than 18 years of age and with either one symptoms of heart failure mentioned by the Framingham criteria^29^ were eligible for this study and were screened by echocardiography. After excluding subjects with LV systolic dysfunction (defined as LV ejection fraction < 45%)^30^, subjects with severe valvular heart disease, and those with atrial fibrillation, totally 238 patients were enrolled for analysis, including 95 cases of diastolic dysfunction and 143 controls. Patients fulfilled the diagnosis of heart failure defined by Framingham criteria^29^ (at least 2 major criteria or 1 major criterion in conjunction with 2 minor criteria) or patients with echocardiographic evidence of diastolic dysfunction would be classified into the case group. The control group was composed of healthy people without either clinical or echocardiographic evidence of heart failure. The flowchart of patient enrollment is shown in Fig. 4. Each patient’s chart was reviewed in detail to collect data on medical history. This research was conducted according to the principles expressed in the Declaration of Helsinki. All participants had given their written informed consents, and the study was approved by the research ethics committee of Taipei Veterans General Hospital.

### Echocardiography and Definition of Diastolic Dysfunction

Echocardiography was performed by trained sonographers using IE33 (Philips Healthcare). LV end-diastolic dimension (LVDd), interventricular septal thickness (IVST) and posterior wall thickness (PWT) were assessed in the parasternal long axis view at end diastole; whereas LA dimension was measured at end systole. We calculated the LV mass by the formula proposed by Devereux *et al*.^31^ with modification^32^: 0.8 × 1.04 × [(LVDd + IVST + PWT)^3^−LVDd^3^] + 0.6. The LV mass index (LVMI) was calculated as the ratio of LV mass to the body surface area (BSA). LV hypertrophy was defined as LVMI greater than 118 g/m^2^ in men, or greater than 108 g/m^2^ in women^33^. LVEF was calculated by using Simpson’s method, with LV end-diastolic and end-systolic volumes acquired in the apical 4-chamber views^34^. Transmitral inflow was measured by pulsed wave blood flow Doppler at the mitral valve leaflet tips in apical 4-chamber view. The peak velocities of early filling (E), atrial filling (A), and the deceleration time of E wave were obtained. The early peak diastolic mitral annulus velocity (e′) was determined by pulsed wave tissue Doppler at LV septum. RVSP was estimated by maximum velocity of tricuspid regurgitation jet measured by continuous wave blood flow Doppler.

Patients were diagnosed to have diastolic dysfunction when either of the following echocardiographic criteria were met:^35^,^36^ (1) septal e′ < 8 and E/e′ ≥ 15, (2) septal e′ < 8 and E/e′ ≥ 8 and E/A < 0.5 and deceleration time of E wave ≥ 280 ms, (3) septal e′ < 8 and E/e′ ≥ 8 and E/A < 0.5 and presence of LV hypertrophy. All patients were followed up for 1 year, or till the occurrence of adverse cardiac events, including heart failure readmission and death.

### Clinical Chemistry and Cardiac Catheterization

Blood samples were obtained after overnight fasting for more than 8 hours. The blood cell count, serum creatinine, sodium, glucose, albumin, lipid profiles, and C-reactive protein were measured by routine laboratory methods. Estimated glomerular filtration rate (eGFR) was calculated by the Modification of Diet in Renal Disease equation for Japanese subjects^37^. Plasma levels of FGF21 were determined by a commercial enzyme-linked immunosorbent assay (ELISA) (R&D Systems, Inc., Minneapolis, MN, USA). The sensitivity was 7 ng/l. Intra- and interassay coefficients were 4.1% and 3.9% respectively. Study subjects were also tested for NT-proBNP by an immunoassay (ELECSYS proBNP, Roche Diagnostics, Germany) using an ELECSYS2010 instrument at the time of enrollment.

The coronary angiograms were interpreted by two experienced interventional cardiologists. Each coronary lesion with a diameter narrowing of more than 50% were considered to be significant stenosis. The central systolic blood pressure (SBP) and central diastolic blood pressure (DBP) were measured at the aortic root level by a 6-French pigtail catheter balanced to the atmospheric pressure. Pulse pressure was defined as the difference between SBP and DBP. The pigtail catheter was also placed into mid-LV cavity to measure the LV end-diastolic pressure (LVEDP) at the onset of the QRS complex. An elevated LV filling pressure was defined as LVEDP > 16 mmHg^13^.

### Statistical Analysis

Clinical, laboratory, and echocardiographic data were presented as mean ± standard deviation for numeric variables, and as counts (percentage) for categorical variables. Patients were grouped according to the presence of diastolic dysfunction. Comparisons between two groups were performed by using Student’s t-test for continuous variables, and using Fisher’s exact test for categorical variables. A Spearman rank correlation test was used to assess the correlation between FGF21 and categorical variables, and a Pearson correlation test was used to assess the correlation between continuous variables. Univariate analysis was performed for FGF21, age, gender, MVD, and clinical chemistries with significant difference between groups. To access the independent effect of FGF21, variables with statistical significance in the univariate analysis were further entered into the multivariate model. The area under the ROC curve (AUC) was used as a measure of the predictive accuracy of FGF21 and NT-pro-BNP. The statistical significance of pairwise comparison between 2 AUCs was tested with the method of DeLong *et al*.^38^. Incidence rate of 1-year adverse events was calculated. Survival curves were generated by the Kaplan–Meier method, and survival among groups was compared by using the log-rank test. Data were analyzed using SPSS version 18.0 (SPSS Inc., Chicago, IL, USA) and MedCalc version 11.4.2.0 (MedCalc Software, Mariakerke, Belgium). A *p*-value < 0.05 was considered to indicate statistical significance.

## Additional Information

**How to cite this article**: Chou, R.-H. *et al*. Circulating Fibroblast Growth Factor 21 is Associated with Diastolic Dysfunction in Heart Failure Patients with Preserved Ejection Fraction. *Sci. Rep.* **6**, 33953; doi: 10.1038/srep33953 (2016).

## Supplementary Material

Supplementary Information

## Figures and Tables

**Table 1:** Baseline characteristics of the study population.

	Total (n = 238)	Control (n = 143)	Diastolic dysfunction (n = 95)	*P*
Age (years)	70.50 ± 13.38	67.87 ± 14.06	74.47 ± 11.23	< 0.001
Male, n (%)	162 (68.07)	109 (76.22)	53 (55.79)	0.001
Body mass index (kg/m^2^) Medical History	25.64 ± 3.73	25.61 ± 3.80	25.66 ± 3.64	0.921
Hypertension, n (%)	180 (75.63)	100 (69.93)	80 (84.21)	0.009
Diabetes, n (%)	80 (33.61)	34 (23.78)	46 (48.42)	<0.001
CKD, n (%)	34 (14.29)	15 (10.49)	19 (20.00)	0.052
NYHA Fc III-IV, n (%)	8 (3.36)	0 (0.00)	8 (8.42)	<0.001
Arrhythmia, n (%)	38 (15.97)	19 (13.29)	19 (20.00)	0.182
Previous MI, n (%)	22 (9.24)	17 (11.89)	5 (5.26)	0.064
PAD, n (%)	37 (15.55)	18 (12.59)	19 (20.00)	0.138
Medications				
Antiplatelets, n (%)	127 (53.36)	82 (57.34)	45 (47.37)	0.132
ACEi or ARB, n (%)	81 (34.03)	40 (27.97)	41 (43.16)	0.018
Beta-blocker, n (%)	66 (27.73)	36 (25.17)	30 (31.58)	0.289
Diuretics, n (%)	62 (26.05)	30 (20.98)	32 (33.68)	0.034
Statins, n (%)	64 (26.89)	37 (25.87)	27 (28.42)	0.666
Laboratory data				
WBC (K/cumm)	7.50 ± 7.20	7.77 ± 9.10	7.09 ± 2.34	0.477
Hemoglobin (g/dL)	12.48 ± 1.87	13.00 ± 1.74	11.70 ± 1.78	<0.001
Platelet (10 K/cumm)	21.19 ± 7.73	21.88 ± 7.54	20.17 ± 7.93	0.094
eGFR (mL/min/1.73 m^2^)	63.04 ± 26.62	68.54 ± 25.69	54.77 ± 25.99	<0.001
Serum sodium (mg/dL)	139.06 ± 4.43	139.24 ± 4.93	138.79 ± 3.57	0.439
Albumin (mg/dL)	4.03 ± 3.81	4.16 ± 4.37	3.89 ± 3.13	0.656
Total cholesterol (mg/dL)	164.48 ± 37.33	162.77 ± 35.48	167.08 ± 40.04	0.389
Triglyceride (mg/dL)	127.21 ± 91.36	126.11 ± 88.91	128.91 ± 95.45	0.819
Fasting glucose (mg/dL)	116.49 ± 42.37	111.14 ± 38.60	124.19 ± 46.43	0.027
CRP (mg/dL)	3.77 ± 7.94	2.74 ± 5.73	4.66 ± 9.41	0.235

CKD = chronic kidney disease, NYHA Fc = the New York Heart Association functional classification, MI = myocardial infarction, PAD = peripheral arterial disease, ACEi = angiotensin-converting-enzyme inhibitor, ARB = angiotensin II receptor blocker, OHA = oral hypoglycemic agent, WBC = white blood cell, eGFR = estimated glomerular filtration rate, CRP = C-reactive protein.

**Table 2:** Log FGF21, log NT-pro-BNP, hemodynamic studies, and echocardiographic parameters of patients grouping by diastolic dysfunction.

	Total (n = 238)	Control (n = 143)	Diastolic dysfunction (n = 95)	*P*
Log FGF-21	2.28 ± 0.41	2.18 ± 0.38	2.42 ± 0.41	<0.001
Log NT-pro-BNP	3.26 ± 0.85	2.95 ± 0.71	3.45 ± 0.88	<0.001
Coronary angiogram				
SVD, n (%)	52 (21.85)	33 (23.07)	19 (20.00)	0.576
MVD, n (%)	101 (42.44)	51 (35.66)	50 (52.63)	0.010
Hemodynamic studies				
Central SBP (mmHg)	154.97 ± 27.69	153.05 ± 25.65	157.72 ± 30.39	0.326
Central DBP (mmHg)	74.78 ± 11.52	76.53 ± 10.18	72.28 ± 12.87	0.038
Pulse pressure (mmHg)	80.19 ± 23.93	76.52 ± 23.46	85.45 ± 23.80	0.029
LVEDP (mmHg)	14.84 ± 5.80	14.25 ± 5.19	15.80 ± 6.60	0.085
Echocardiography				
LA dimension (mm)	40.20 ± 7.39	38.55 ± 6.95	42.62 ± 7.39	<0.001
IVST (mm)	11.07 ± 3.64	10.50 ± 2.38	11.91 ± 4.83	0.003
LVDd (mm)	48.39 ± 7.70	48.14 ± 6.37	48.75 ± 9.33	0.577
PWT (mm)	10.67 ± 2.22	10.45 ± 1.99	11.00 ± 2.50	0.061
LV mass index (g/m^2^)	117.74 ± 52.02	106.53 ± 36.75	134.02 ± 65.25	<0.001
LV ejection fraction (%)	56.04 ± 7.97	56.82 ± 6.54	54.90 ± 9.61	0.093
e′ velocity (cm/s)	6.08 ± 2.33	7.11 ± 2.29	4.63 ± 1.44	<0.001
E/e′ ratio	15.66 ± 8.77	10.65 ± 2.68	22.58 ± 9.53	<0.001
E/A ratio	0.90 ± 0.39	0.89 ± 0.35	0.91 ± 0.45	0.632
Deceleration time (msec)	229.96 ± 60.68	220.43 ± 50.51	243.69 ± 70.95	0.007
RVSP (mmHg)	34.91 ± 12.84	31.92 ± 10.58	38.60 ± 14.39	<0.001

SVD = single vessel disease, MVD = multiple vessels disease, SBP = systolic blood pressure, DBP = diastolic blood pressure, LVEDP = left ventricular end-diastolic pressure, LA = left atrial, IVST = interventricular septal thickness, LVDd = left ventricular end-diastolic dimension, PWT = posterior wall thickness, LV = left ventricular, RVSP = right ventricular systolic pressure.

**Table 3:** Correlation coefficients of log FGF21 and log NT-pro-BNP for the association with clinical and echocardiographic variables.

Variable	Log FGF21		Log NT-pro-BNP	
	R	*P value*	R	*P value*
Log FGF21	—	—	0.493	<0.001
Log NT-pro-BNP	0.493	<0.001	—	—
Age (years)	0.119	0.067	0.260	<0.001
Gender (male = 1)	−0.140	0.030	−0.035	0.595
Body mass index (kg/m^2^)	0.052	0.428	−0.192	0.003
Hypertension	0.285	<0.001	0.239	<0.001
Diabetes	0.220	0.001	0.282	<0.001
NYHA class III-IV HF	0.178	0.006	0.253	<0.001
Coronary artery disease	0.139	0.032	0.292	<0.001
Peripheral artery disease	0.152	0.019	0.243	<0.001
Laboratory data				
Hemoglobin (mg/dL)	−0.324	<0.001	−0.424	<0.001
eGFR (mL/min/1.73m^2^)	−0.472	<0.001	−0.527	<0.001
Total cholesterol (mg/dL)	0.018	0.787	−0.002	0.970
Triglyceride (mg/dL)	0.281	<0.001	0.006	0.924
FBS (mg/dL)	0.191	0.004	0.226	0.001
CRP (mg/dL)	0.181	0.076	0.230	0.023
Hemodynamic studies				
Central DBP (mmHg)	−0.169	0.045	−0.185	0.028
Pulse pressure (mmHg)	0.086	0.301	0.197	0.019
LVEDP (mmHg)	0.253	<0.001	0.205	0.004
Echocardiography				
LA dimension (mm)	0.259	<0.001	0.344	<0.001
IVST (mm)	0.254	<0.001	0.282	<0.001
LV mass index (g/m^2^)	0.268	<0.001	0.425	<0.001
e′ velocity (cm/s)	−0.283	<0.001	−0.292	<0.001
E/e′ ratio	0.321	<0.001	0.401	<0.001
Deceleration time (msec)	−0.060	0.372	−0.113	0.088
RVSP (mmHg)	0.140	0.043	0.326	<0.001

NYHA Fc = the New York Heart Association functional classification, eGFR = estimated glomerular filtration rate, FBS = fasting blood sugar, CRP = C-reactive protein, DBP = diastolic blood pressure, LVEDP = left ventricular end-diastolic pressure, LA = left atrial, IVST = interventricular septal thickness, LV = left ventricular, RVSP = right ventricular systolic pressure.

**Table 4:** Logistic regression analysis using diastolic dysfunction as dependent variable.

Variable	Univariate		Model 1*		Model 2^†^	
	OR (95% CI)	*P value*	OR (95% CI)	*P value*	OR (95% CI)	*P value*
Diastolic dysfunction: broad definition (n = 95)						
Log FGF21	4.71 (2.31–9.60)	<0.001	4.25 (2.00–9.05)*	<0.001*	2.97 (1.28–6.91)^†^	0.012^†^
Log NT-pro-BNP	3.19 (2.19–4.64)	<0.001	3.07 (2.06–4.58)*	<0.001*	2.85 (1.83–4.44)^†^	<0.001^†^
Age	1.04 (1.02–1.06)	<0.001	1.05 (1.02–1.07)	<0.001	1.04 (1.02–1.07)	0.001
Gender	0.39 (0.23–0.69)	0.001	0.36 (0.19–0.67)	0.001	0.32 (0.17–0.61)	<0.001
MVD	2.00 (1.18–3.40)	0.010			1.72 (0.95–3.11)	0.075
eGFR	0.98 (0.97–0.99)	<0.001			0.99 (0.98–1.00)	0.179
FBS	1.01 (0.99–1.03)	0.052				
Diastolic dysfunction: strict definition (n = 48)						
Log FGF21	6.01 (2.35–15.39)	<0.001	5.84 (2.27–14.99)*	<0.001*	3.73 (1.13–12.25)^†^	0.030^†^
Log NT-pro-BNP	2.07 (1.38–3.12)	<0.001	2.07 (1.38–3.12)*	<0.001*	1.45 (0.86–2.45)^†^	0.164^†^
Age	1.00 (0.97–1.02)	0.729	0.99 (0.96–1.02)	0.561	0.99 (0.96–1.03)	0.678
Gender	0.59 (0.28–1.25)	0.167	0.69 (0.31–1.50)	0.343	0.72 (0.32–1.62)	0.423
MVD	2.30 (1.10–4.79)	0.026			1.14 (0.50–2.60)	0.753
eGFR	0.98 (0.96–0.99)	0.002			0.99 (0.97–1.01)	0.202
FBS	1.01 (1.00–1.02)	0.018			1.01 (1.00–1.01)	0.151

^*^Adjusted age and gender.

^†^Adjusted age, gender, and statistically significant variables in univariate analysis.

MVD = multiple vessel disease, eGFR = estimated glomerular filtration rate, FBS = fasting blood sugar.
