# Supplementary material for: Circulating Fibroblast Growth Factor 21 is Associated with Diastolic Dysfunction in Heart Failure Patients with Preserved Ejection Fraction
Source: Sci Rep. 2016 Sep 21;6:33953. doi: 10.1038/srep33953 (PMC5030655; doi:10.1038/srep33953)
Supplement: Supplementary Information [file srep33953-s1.doc]

**SUPPLEMENT**

**Circulating Fibroblast Growth Factor 21 is Associated with Diastolic Dysfunction in Heart Failure Patients with Preserved Ejection Fraction**

**Ruey-Hsing Chou,^1,6,7^ Po-Hsun Huang,^1,6,7^* Chien-Yi Hsu,^6,7,9,10^ Chun-Chin Chang^1,6,7^, Hsin-Bang Leu,^1,2,6,7^ Chin-Chou Huang,^1,3,6,8^ Jaw-Wen Chen,^1,4,5,6,8^, Shing-Jong Lin^1,4,6,7,9^**

^1^ Division of Cardiology, Department of Medicine Taipei Veterans General Hospital, Taipei, 11217, Taiwan;

^2^ Healthcare and Management Center Taipei Veterans General Hospital, Taipei, 11217, Taiwan;

^3^ Department of Medical Education Taipei Veterans General Hospital, Taipei, 11217, Taiwan;

^4^ Department of Medical Research Taipei Veterans General Hospital, Taipei, 11217, Taiwan;

^5^ Division of Clinical Research Taipei Veterans General Hospital, Taipei, 11217, Taiwan;

^6^ Cardiovascular Research Center, Taipei Veterans General Hospital, Taipei, 11217, Taiwan;

^7^ Institute of Clinical Medicine, National Yang-Ming University, Taipei, 11217, Taiwan;

^8^ Institute of Pharmacology, National Yang-Ming University, Taipei, 11217, Taiwan;

^9^ Department of Internal Medicine, College of Medicine, Taipei Medical University, Taipei, 11031, Taiwan;

^10^ Division of Cardiology and Cardiovascular Research Center, Department of Internal Medicine, Taipei Medical University Hospital, Taipei, 11031, Taiwan.

**Supplement Table 1.** Linear regression analysis examining the association between various parameters and E/e', LVEDP.

| Variable | **Univariate** | |  | **Model 1*** | |  | **Model 2^✝^** | |
| --- | --- | --- | --- | --- | --- | --- | --- | --- |
|  | **Std β** | ***P value*** |  | **Std β** | ***P value*** |  | **Std β** | ***P value*** |
| **Dependent variable: E/e’** |  |  |  |  |  |  |  |  |
| Log FGF21 | 0.321 | <0.001 |  | 0.275* | <0.001* |  | 0.198^✝^ | 0.007^✝^ |
| Log NT-pro-BNP | 0.401 | <0.001 |  | 0.352* | <0.001* |  | 0.258^✝^ | 0.001^✝^ |
| Age | 0.192 | 0.004 |  | 0.177 | 0.005 |  | 0.176 | 0.009 |
| Gender (male=1) | -0.208 | 0.002 |  | -0.189 | 0.004 |  | -0.193 | 0.003 |
| MVD | 0.125 | 0.022 |  |  |  |  | 0.062 | 0.340 |
| eGFR | -0.255 | <0.001 |  |  |  |  | -0.082 | 0.268 |
| FBS | 0.146 | 0.031 |  |  |  |  | 0.113 | 0.083 |
| **Dependent variable: LVEDP** |  |  |  |  |  |  |  |  |
| Log FGF21 | 0.253 | <0.001 |  | 0.297* | <0.001* |  | 0.200^✝^ | 0.011^✝^ |
| Log NT-pro-BNP | 0.205 | 0.004 |  | 0.286* | <0.001* |  | 0.149^✝^ | 0.082^✝^ |
| Age | -0.189 | 0.008 |  | -0.236 | 0.001 |  | -0.234 | 0.001 |
| Gender (male=1) | 0.035 | 0.627 |  | 0.090 | 0.190 |  | 0.077 | 0.257 |
| MVD | 0.094 | 0.187 |  |  |  |  |  |  |
| eGFR | -0.208 | 0.003 |  |  |  |  | -0.153 | 0.053 |
| FBS | 0.253 | <0.001 |  |  |  |  | 0.162 | 0.020 |
| *= adjusted age and gender  ^✝^= adjusted age, gender, and statistically significant variables in univariate analysis  LVEDP= left ventricular end-diastolic pressure,  MVD= multiple vessel disease,  eGFR= estimated glomerular filtration rate,  FBS= fasting blood sugar | | | | | | | | |

**Supplement Table 2.** Logistic regression analysis using diastolic dysfunction as dependent variable, and grouping patients by FGF21 levels

| Variable | **Univariate** | | |  | **Model 1*** | |  | | **Model 2^✝^** | |
| --- | --- | --- | --- | --- | --- | --- | --- | --- | --- | --- |
|  | **OR (95% CI)** | | ***P value*** |  | **OR (95% CI)** | ***P value*** |  | | **OR (95% CI)** | ***P value*** |
| **Diastolic dysfunction: broad definition (n=95)** | | | | | | | | | | |
| FGF21 level^#^ | |  |  |  |  |  |  |  | |  |
| *Quartile 1* | | *Reference* |  |  | *Reference* |  |  | *Reference* | |  |
| *Quartile 2* | | 1.56 (0.69-3.51) | 0.281 |  | 1.22 (0.52-2.86) | 0.643 |  | 1.24 (0.52-2.97) | | 0.622 |
| *Quartile 3* | | 2.99 (1.35-6.52) | 0.007 |  | 2.11 (0.92-4.85) | 0.077 |  | 2.03 (0.86-4.80) | | 0.106 |
| *Quartile 4* | | 4.30 (1.96-9.44) | <0.001 |  | 3.64 (1.59-8.39) | 0.002 |  | 2.47 (0.99-6.14) | | 0.052 |
| Age | | 1.04 (1.02-1.06) | <0.001 |  | 1.05 (1.02-1.07) | <0.001 |  | 1.04 (1.02-1.07) | | 0.002 |
| Gender | | 0.39 (0.23-0.69) | 0.001 |  | 0.37 (0.20-0.68) | 0.001 |  | 0.33 (0.17-0.62) | | 0.001 |
| MVD | | 2.00 (1.18-3.40) | 0.010 |  |  |  |  | 1.79 (0.98-3.27) | | 0.058 |
| eGFR | | 0.98 (0.97-0.99) | <0.001 |  |  |  |  | 0.99 (0.98-1.00) | | 0.086 |
| FBS | | 1.01 (0.99-1.03) | 0.052 |  |  |  |  |  | |  |
| **Diastolic dysfunction: strict definition (n=48)** | | | | | | | | | | |
| FGF21 level^#^ | |  |  |  |  |  |  | |  |  |
| *Quartile 1* | | *Reference* |  |  | *Reference* |  |  | | *Reference* |  |
| *Quartile 2* | | 2.08 (0.61-7.12) | 0.244 |  | 1.21 (0.34-4.34) | 0.766 |  | | 0.96 (0.26-3.54) | 0.951 |
| *Quartile 3* | | 2.08 (0.61-7.12) | 0.244 |  | 1.26 (0.36-4.44) | 0.721 |  | | 1.05 (0.29-3.79) | 0.943 |
| *Quartile 4* | | 6.69 (2.30-19.44) | <0.001 |  | 3.83 (1.28-11.47) | 0.016 |  | | 1.92 (0.53-6.93) | 0.321 |
| Age | | 1.00 (0.97-1.02) | 0.729 |  | 1.00 (0.97-1.03) | 0.760 |  | | 1.00 (0.96-1.03) | 0.762 |
| Gender | | 0.59 (0.28-1.25) | 0.167 |  | 0.67 (0.31-1.45) | 0.305 |  | | 0.67 (0.30-1.51) | 0.671 |
| MVD | | 2.30 (1.10-4.79) | 0.026 |  |  |  |  | | 1.21 (0.53-2.73) | 0.650 |
| eGFR | | 0.98 (0.96-0.99) | 0.002 |  |  |  |  | | 0.98 (0.97-1.00) | 0.061 |
| FBS | | 1.01 (1.00-1.02) | 0.018 |  |  |  |  | | 1.01 (1.00-1.01) | 0.128 |
| ^#^ In *Quartile 1* (n=60), the FGF21 level was 62.92 ±20.11 ng/l. (presented as mean ±SD),  In *Quartile 2* (n=59), the FGF21 level was 143.24 ±25.58 ng/l.  In *Quartile 3* (n=59), the FGF21 level was 236.97 ±39.15 ng/l.  In *Quartile 4* (n=60), the FGF21 level was 765.48 ±490.62 ng/l.  *= adjusted age and gender  ^✝^= adjusted age, gender, and statistically significant variables in univariate analysis  MVD= multiple vessel disease, eGFR= estimated glomerular filtration rate, FBS= fasting blood sugar,  SD= standard deviation | | | | | | | | | | |
